# Correction: Tasci et al. RadWise: A Rank-Based Hybrid Feature Weighting and Selection Method for Proteomic Categorization of Chemoirradiation in Patients with Glioblastoma. *Cancers* 2023, *15*, 2672

**DOI:** 10.3390/cancers16152744

**Published:** 2024-08-01

**Authors:** Erdal Tasci, Sarisha Jagasia, Ying Zhuge, Mary Sproull, Theresa Cooley Zgela, Megan Mackey, Kevin Camphausen, Andra Valentina Krauze

**Affiliations:** Radiation Oncology Branch, Center for Cancer Research, National Cancer Institute, National Institutes of Health, Building 10, Bethesda, MD 20892, USA

In the original publication [1], there was a mistake shown in Tables 1–8 and Figures 3–5 and 7 as published. The pre/post-categorization information of four patients in our proteomic dataset was incorrectly labeled, requiring the proteomic analysis to be repeated. We repeated our analyses depending on the newly constructed, corrected, and normalized dataset. The newly obtained results are given in the corrected Table 1, Table 2, Table 3, Table 4, Table 5, Table 6, Table 7 and Table 8 and Figure 3, Figure 4 and Figure 5 and Figure 7 below.

**References**

59. Zeng, S.; Li, W.; Ouyang, H.; Xie, Y.; Feng, X.; Huang, L. A Novel Prognostic Pyroptosis-Related Gene Signature Correlates to Oxidative Stress and Immune-Related Features in Gliomas. *Oxid. Med. Cell. Longev.* **2023**, *2023*, 4256116. https://doi.org/10.1155/2023/4256116.

The repeat analysis has resulted in superior results as compared to the previous analysis with the best ACC% now 96.364, which was obtained with the Logistic Regression Model, and the minimum weight of 10. The selected number of features is now 8. Best Feature (Biomarker) Set is as follows: ‘K2C5’, ‘MIC-1’, ‘CSPG3’, ‘GFAP’, ‘STRATIFIN’, ‘Cystatin M’, ‘Keratin-1’ and ‘Proteinase-3’. All places in the manuscript text where the new results have resulted in a numerical change e.g., ACC, AUC, have been corrected to reflect the new findings in the updated tables. With this correction, the order of some references has been adjusted accordingly. The authors state that the scientific conclusions are unaffected. This correction was approved by the Academic Editor. The original publication has also been updated.

## Figures and Tables

**Figure 3:**
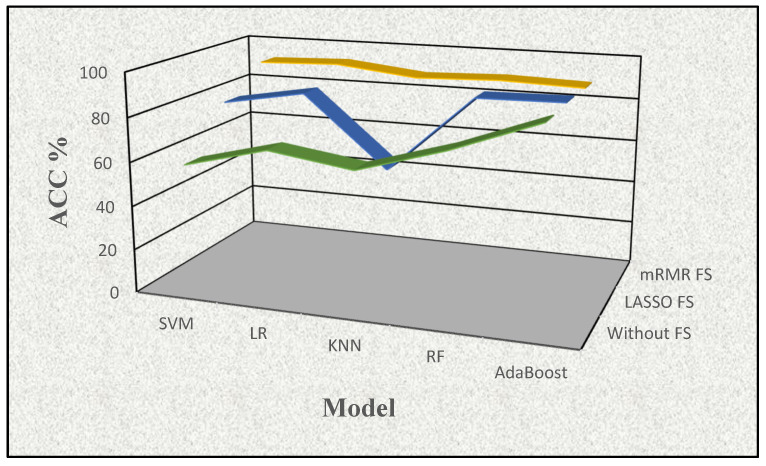
The visualization of the effects of the feature selection procedures with accuracy (ACC%) determined by a supervised learning method in conjunction with the feature selection approach (mRMR FS (yellow), LASSO FS (blue), and no FS (green)).

**Figure 4:**
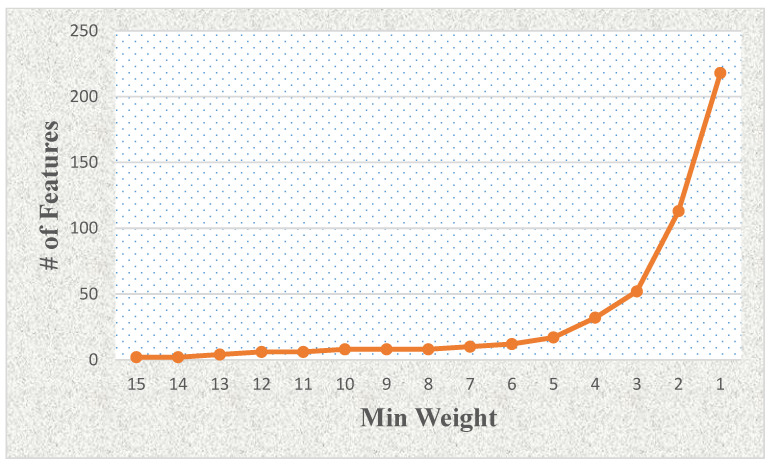
The effects of the number of features related to the minimum weight value using LASSO and mRMR-based feature selection with weighting methods.

**Figure 5:**
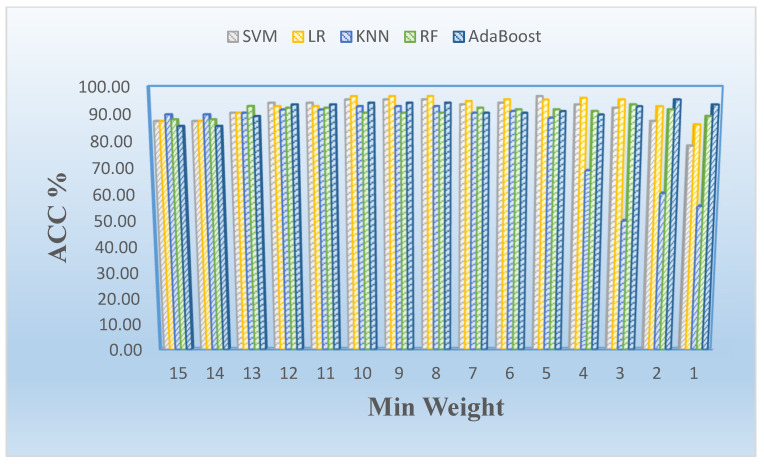
Mean accuracy rate (ACC) vs. minimum weight stratified by model employed in analysis.

**Figure 7:**
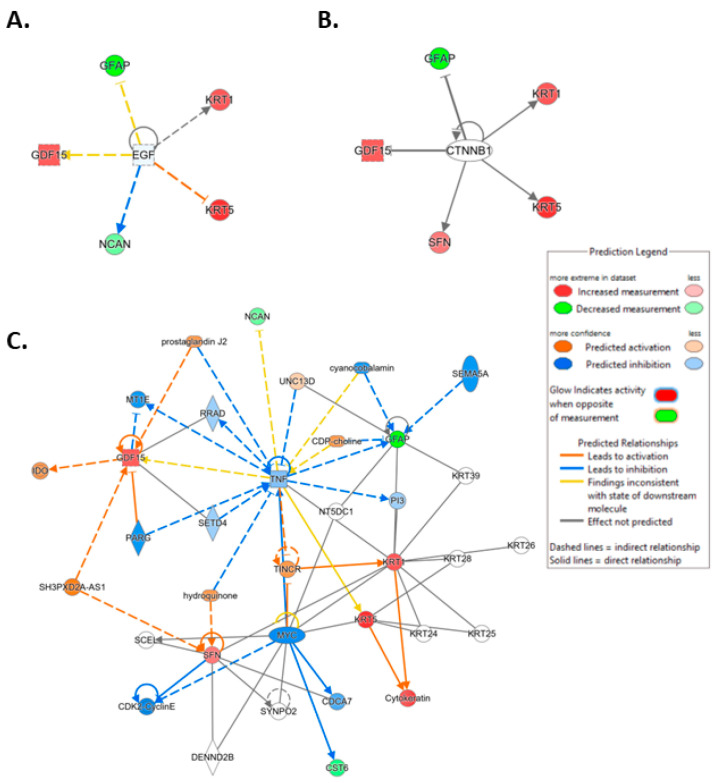
Ingenuity pathway analysis (IPA) carried out on April 5, 2023, illustrating linkage of the identified protein features to the top 2 upstream mediators (Supplementary Data Table S2). (**A**) Epidermal growth factor (EGF) (*p*-value of overlap 2.53 × 10^−7^). (**B**) Catenin beta 1 (CTNNB1) (*p*-value of overlap 2.41 × 10^−6^). (**C**) IPA-generated merged network for the 8 ML-identified proteins using the disease classification brain cancer.

**Table 1:** Accuracy rates: Five supervised learning models with or without feature selection. Color changes from red to green display performance results from the lowest (red) to the highest values (green).

ML-ACC	Without FS	LASSO FS	mRMR FS
**SVM**	57.860	78.674	91.515
**LR**	67.633	85.341	92.708
**KNN**	62.197	53.068	88.466
**RF**	73.826	88.466	89.659
**AdaBoost**	88.409	89.072	88.447

**Table 2:** Performance results (i.e., ACC%) using only LASSO-based feature selection and weighting methods. Color changes from red to green display performance results from the lowest (red) to the highest values (green). The bold value indicates the best result.

k	# of Features	SVM	LR	KNN	RF	AdaBoost
**5**	11	93.314	92.083	82.386	91.496	91.477
**4**	26	89.640	93.314	62.197	93.939	90.890
**3**	44	89.053	96.363	46.345	92.121	93.939
**2**	90	85.985	92.064	60.379	91.496	93.901
**1**	197	76.269	85.966	54.868	87.841	87.822

**Table 3:** Performance results (i.e., ACC%) using only mRMR-based feature selection and weighting methods. Color changes from red to green display performance results from the lowest (red) to the highest (green) values. The bold value indicates the best result.

k	# of Features	SVM	LR	KNN	RF	AdaBoost
**5**	5	86.004	88.428	87.235	87.841	87.197
**4**	7	90.890	92.708	90.265	91.496	91.496
**3**	8	95.152	96.364	92.708	90.871	93.920
**2**	11	92.708	96.364	92.689	90.284	94.508
**1**	34	92.102	92.102	87.254	92.121	90.871

**Table 4:** Mean performance results (i.e., ACC %, CV = 5) determined using both LASSO and mRMR-based feature selection with weighting methods. Color changes from red to green display performance results from the lowest (red) to the highest values (green). The bold value indicates the best result.

k	# of Features	SVM	LR	KNN	RF	AdaBoost
**15**	2	87.216	87.216	89.659	87.841	85.379
**14**	2	87.216	87.216	89.659	87.841	85.379
**13**	4	90.265	90.265	90.284	92.727	89.034
**12**	6	93.920	92.708	91.477	92.102	93.314
**11**	6	93.920	92.708	91.477	92.102	93.314
**10**	8	95.152	96.364	92.708	90.265	93.920
**9**	8	95.152	96.364	92.708	90.265	93.920
**8**	8	95.152	96.364	92.708	90.265	93.920
**7**	10	93.333	94.546	90.284	92.102	90.284
**6**	12	93.939	95.152	90.909	91.496	90.246
**5**	17	96.345	95.114	88.447	91.496	90.871
**4**	32	93.295	95.739	68.921	90.890	89.640
**3**	52	92.121	95.151	49.962	93.333	92.670
**2**	113	87.216	92.670	60.379	91.496	95.152
**1**	218	78.087	85.966	55.492	89.053	93.314

**Table 5:** The standard deviation of performance results (i.e., ACC %, CV = 5) determined using both LASSO and mRMR-based feature selection with weighting methods. Color changes from red to green display performance results from the lowest (red) to the highest values (green).

k	# of Features	SVM	LR	KNN	RF	AdaBoost
**15**	2	5.175	4.408	4.072	4.232	2.191
**14**	2	5.175	4.408	4.072	4.232	2.191
**13**	4	4.423	4.821	4.425	4.924	2.384
**12**	6	5.060	5.269	3.509	4.088	3.515
**11**	6	5.060	5.269	3.509	4.088	3.515
**10**	8	3.636	4.454	5.269	3.496	4.272
**9**	8	3.636	4.454	5.269	3.496	4.272
**8**	8	3.636	4.454	5.269	3.496	4.272
**7**	10	4.848	5.555	4.425	4.088	4.425
**6**	12	4.285	3.636	6.357	3.995	3.526
**5**	17	2.966	2.444	3.476	4.827	5.400
**4**	32	6.177	4.105	6.384	4.259	1.442
**3**	52	4.535	2.424	7.329	4.020	2.469
**2**	113	4.807	1.557	4.954	4.430	4.924
**1**	218	4.720	3.133	5.559	3.031	3.515

**Table 6:** Performance results without employing feature selection and feature weighting. Color changes from red to green display performance results from the lowest (red) to the highest values (green).

ML	ACC%	AUC	F1	PRE	REC	SPEC
**SVM**	57.860	0.415	0.518	0.698	0.515	0.690
**LR**	67.633	0.755	0.676	0.681	0.681	0.673
**KNN**	62.197	0.647	0.581	0.662	0.527	0.722
**RF**	73.826	0.808	0.744	0.768	0.746	0.737
**AdaBoost**	88.409	0.951	0.886	0.882	0.893	0.873

**Table 7:** Performance results employing LASSO and mRMR-based feature selection with weighting operation. Color changes from red to green display performance results from the lowest (red) to the highest values (green).

ML	ACC%	AUC	F1	PRE	REC	SPEC
**SVM**	95.152	0.989	0.949	0.975	0.928	0.976
**LR**	96.364	0.987	0.964	0.963	0.965	0.965
**KNN**	92.708	0.965	0.930	0.929	0.932	0.923
**RF**	90.265	0.978	0.902	0.885	0.928	0.876
**AdaBoost**	93.920	0.979	0.941	0.941	0.942	0.935

The best ACC% is 96.364, which was obtained with the Logistic Regression Model, and the minimum weight is 10. Selected Number of Features: 8. Best Feature (Biomarker) Set is as follows: ‘K2C5’, ‘MIC-1’, ‘CSPG3’, ‘GFAP’, ‘Proteinase-3’, ‘STRATIFIN’, ‘Cystatin M’, and ‘Keratin-1’ [59].

**Table 8:** Overview of the identified proteomic biomarkers illustrating the biological relevance to glioma.

Entrez Gene Symbol	Target Full Name	Biological Relevance to Glioma
K2C5	Keratin, type II cytoskeletal 5	Yes, evolving biomarker/target [61]
Keratin-1	Keratin, type II cytoskeletal 1	Yes, evolving biomarker/target [61]
STRATIFIN (SFN)	14-3-3 protein sigma	Yes, tumor suppressor gene expression pattern correlates with glioma grade and prognosis [62]
MIC-1 (GDF15)	Growth/differentiation factor 15	Yes, biomarker, novel immune checkpoint [63]
GFAP	Glial fibrillary acidic protein	Yes, evolving biomarker/target [64]
CSPG3 (NCAN)	Neurocan core protein	Yes, glycoproteomic profiles of GBM subtypes, differential expression versus control tissue [65]
Cystatin M (CST6)	Cystatin M	Yes, cell type-specific expression in normal brain and epigenetic silencing in glioma [66]
Proteinase-3 (PRTN3)	Proteinase-3	Yes, evolving role, may relate to pyroptosis, oxidative stress and immune response [59]

## References

[1] Tasci E., Jagasia S., Zhuge Y., Sproull M., Cooley Zgela T., Mackey M., Camphausen K., Krauze A.V. (2023). RadWise: A Rank-Based Hybrid Feature Weighting and Selection Method for Proteomic Categorization of Chemoirradiation in Patients with Glioblastoma. Cancers.

