# Supplementary figures and images for: RadWise: A Rank-Based Hybrid Feature Weighting and Selection Method for Proteomic Categorization of Chemoirradiation in Patients with Glioblastoma
Source: Cancers (Basel). 2023 May 9;15(10):2672. doi: 10.3390/cancers15102672 (PMC10216128; doi:10.3390/cancers15102672)

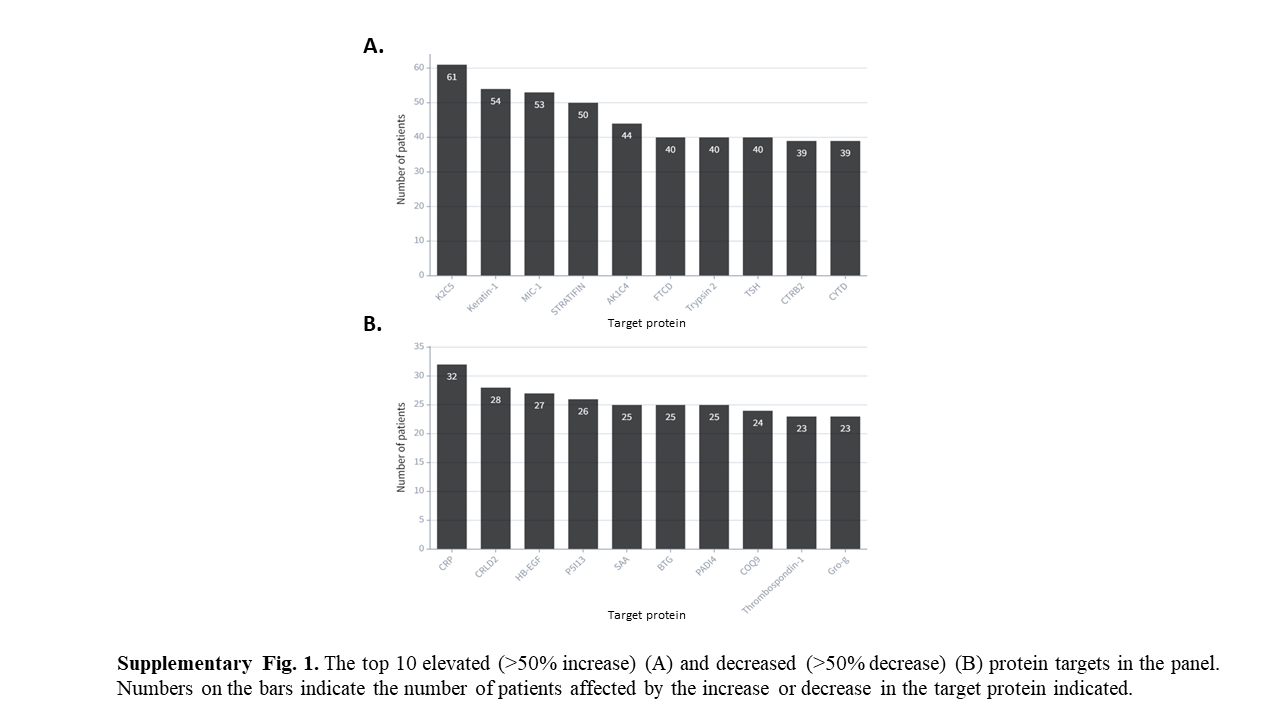

Supplement: Supplementary file 1 [file cancers-15-02672-s001.zip › Figure S1.PNG]
